# The Heme Metabolite Carbon Monoxide Facilitates KSHV Infection by Inhibiting TLR4 Signaling in Endothelial Cells

**DOI:** 10.3389/fmicb.2017.00568

**Published:** 2017-04-03

**Authors:** Sara Botto, Jean K. Gustin, Ashlee V. Moses

**Affiliations:** Vaccine and Gene Therapy Institute, Oregon Health and Science University, PortlandOR, USA

**Keywords:** KSHV, carbon monoxide, CO, HO-1, heme oxygenase-1, TLR4, innate immunity

## Abstract

Kaposi sarcoma herpesvirus (KSHV) is the etiologic agent of Kaposi sarcoma (KS) and certain rare B cell lymphoproliferative disorders. KSHV infection of endothelial cells (EC) *in vitro* increases expression of the inducible host-encoded enzyme heme oxygenase-1 (HO-1), which is also strongly expressed in KS tumors. HO-1 catalyzes the rate-limiting step in the conversion of heme into iron, biliverdin and the gasotransmitter carbon monoxide (CO), all of which share anti-apoptotic, anti-inflammatory, pro-survival, and tumorigenic activities. Our previous work has shown that HO-1 expression in KSHV-infected EC is characterized by a rapid yet transient induction at early times post-infection, followed by a sustained upregulation co-incident with establishment of viral latency. These two phases of expression are independently regulated, suggesting distinct roles for HO-1 in the virus life cycle. Here, we investigated the role of HO-1 during acute infection, prior to the onset of viral gene expression. The early infection phase involves a series of events that culminate in delivery of the viral genome to the nucleus. Primary infection also leads to activation of host innate immune effectors, including the pattern recognition receptor TLR4, to induce an antiviral response. It has been shown that TLR4-deficient EC are more susceptible to KSHV infection than wild-type controls, suggesting an important inhibitory role for TLR4 in the KSHV life cycle. TLR4 signaling is in turn subject to regulation by several virus-encoded immune evasion factors. In this report we identify HO-1 as a host protein co-opted by KSHV as part of a rapid immune evasion strategy. Specifically, we show that early HO-1 induction by KSHV results in increased levels of endogenous CO, which functions as a TLR4 signaling inhibitor. In addition, we show that CO-mediated inhibition of TLR4 signaling leads to reduced expression of TLR4-induced antiviral genes, thus dampening the host antiviral response and facilitating KSHV infection. Conversely, inhibition of HO-1 activity decreases intracellular CO, enhances the host antiviral response and inhibits KSHV infection. In conclusion, this study identifies HO-1 as a novel innate immune evasion factor in the context of KSHV infection and supports HO-1 inhibition as a viable therapeutic strategy for KS.

## Introduction

Kaposi sarcoma herpesvirus (KSHV), a tumorigenic human gamma-2 herpesvirus, is the etiologic agent of Kaposi sarcoma (KS) (Moore and Chang, 1998) and the lymphoproliferative disorders known as primary effusion lymphoma (PEL) and plasmablastic multicentric Castleman disease (MCD) (Moore and Chang, 1998; Cesarman and Mesri, 2007). KSHV infection of lymphatic endothelial cells (LEC) *in vitro* results in induction of the stress-inducible host enzyme heme oxygenase-1 (HO-1). KS spindle cells in biopsy tissue also strongly express HO-1, suggesting that the enzyme may play a role in tumorigenesis, as has been shown for HO-1 in other cancers (McAllister et al., 2004; Chau, 2015). *In vitro*, KSHV induction of HO-1 occurs in two distinct phases, a transient phase upon acute infection and a sustained phase coincident with the establishment of viral latency. The initial phase of HO-1 upregulation is independent of *de novo* viral gene expression, suggesting that virion components contribute to initial induction (Botto et al., 2015). This observation is supported by an independent study demonstrating induction of HO-1 early post KSHV infection via an NRF2-dependent mechanism in response to infection-associated oxidative stress (Gjyshi et al., 2014).

Heme oxygenase-1 enzymatic activity catalyzes the degradation of heme into carbon monoxide (CO), ferrous iron, and biliverdin, which is subsequently converted to bilirubin (Ryter and Choi, 2009). HO-1 is ubiquitously expressed, albeit at low levels in most cell types, but is rapidly induced in response to diverse cellular stressors, as well as being strongly expressed in several types of cancer (Ryter and Choi, 2015). HO-1 is thought to contribute to cytoprotection and proliferation through the catabolism of heme, a pro-oxidant molecule that is cytotoxic in excess, into metabolites with anti-oxidant, pro-angiogenic, anti-apoptotic, and anti-inflammatory activities (Ryter and Tyrrell, 2000; Ryter et al., 2006; Ryter and Choi, 2009). Of particular relevance to the current study, the endogenous gasotransmitter CO participates in intracellular signaling pathways that culminate in anti-inflammatory, anti-coagulative and pro-survival outcomes (Liu et al., 2002; Pae et al., 2004; Otterbein et al., 2005; Nakahira et al., 2006; Ryter and Choi, 2009; Ruan et al., 2014; Yang et al., 2014; Xue and Habtezion, 2014; Riquelme et al., 2015). The discovery of compounds categorized as “carbon monoxide releasing molecules” (CORM) has allowed for further mechanistic insight into the role of CO in biological systems (Ryter and Choi, 2015). Interestingly, CO is able to inhibit innate immune responses and in particular to block TLR4 signaling by preventing the interaction between TLR4 and its adaptor proteins MyD88 and TRIF (Nakahira et al., 2006; Wang et al., 2009; Chi et al., 2014). This down-modulatory effect reveals an important anti-inflammatory role for CO in pathogen-exposed cells and suggests that CO could contribute to immune evasion by pathogens such as KSHV that induce HO-1. Innate immunity represents the first layer of host defense against pathogens, and appropriate sensing of non-self is critical for initiating an effective immune response (O’Neill et al., 2013). Early responses to microbial pathogens are typically initiated by a class of immune sensors known as pattern recognition receptors (PRRs). The Toll-like receptors (TLRs) are a subclass of PRRs that detect a broad range of pathogens (Boehme and Compton, 2004). KSHV activates multiple TLRs including TLR9, which recognizes unmethylated CpG motifs of double-stranded DNA (dsDNA) (West et al., 2011), TLR3, which senses virus replication intermediates such as dsRNA (West and Damania, 2008) and TLR4, which is most well-known for recognizing Gram-negative bacterial lipopolysaccharide (LPS) (Lagos et al., 2008). The significance of the balance between KSHV and host immunity is underscored by the increased development of KSHV-associated malignancies in the setting of immunodeficiency (Feng et al., 2013). Viruses adopt a variety of mechanisms to evade the host immune response, and KSHV encodes several proteins and micro-RNAs that target critical host components of both the innate and the adaptive immune system (Feng et al., 2013; Brulois and Jung, 2014). Although the KSHV component(s) that activate TLR4 signaling remain to be identified, UV-inactivated KSHV induces TLR4 signaling, suggesting that one or more viral glycoproteins are responsible (Lagos et al., 2008). Downregulation of TLR4 in endothelial cells, the precursors of KS tumor cells, leads to an enhanced susceptibility to KSHV infection (Lagos et al., 2008), suggesting an important role for this receptor in the regulation of endothelial infection.

In the present study, we have explored the role of CO, a gasotransmitter endogenously generated by the enzymatic activity of HO-1, in modulating TLR4 signaling during *de novo* KSHV infection of endothelial cells, via the use of an intracellular CO sensor and a small molecule inhibitor of HO-1 activity. We find that intracellular CO levels are enhanced by KSHV infection and that CO is capable of inhibiting TLR4 signaling to promote KSHV infection.

## Materials and Methods

### Cells and Viruses

Primary adult human LEC were obtained from Lonza (Allendale, NJ, USA). Immortalized LEC (iLEC) were generated from primary LEC via transduction with a retroviral vector expressing HPV16 E6/E7, as previously described (Moses et al., 1999; McAllister and Moses, 2007). iLEC were cultured in EGM-2 prepared by supplementing basal medium (EBM-2; Lonza) with 10% fetal bovine serum (FBS), penicillin-streptomycin–L-glutamine (PSG; HyClone, Logan, UT, USA), and additional EC growth supplements (EGM-2MV Bullet kit; Lonza). HeLa cells were cultured in DMEM with 10% FBS and PSG. The epithelial iSLK cell line (generously provided by Dr. Rolf Renne, University of Florida, Gainesville, FL, USA with the kind permission of Dr. Jae Jung, University of Southern California, Los Angeles, CA, USA and Dr. Don Ganem, Novartis Institutes for BioMedical Research) was used to produce stocks of recombinant KSHV. iSLK cells were grown in DMEM supplemented with 10% FBS, PSG, puromycin (1 μg/ml), neomycin (200 μg/ml), and hygromycin (1.5 mg/ml). The recombinant KSHV BAC clone BAC16, derived from rKSHV.219 and kindly provided by Dr. Jung, was used in all KSHV infection experiments (Brulois et al., 2012). To produce stocks of KSHV-BAC16, iSLK cells harboring the BAC16 genome were treated with doxycycline (1 μM) and sodium butyrate (3 mM) for 72 h. Supernatants were then collected, clarified by low-speed centrifugation, transferred to ultracentrifuge tubes, underlaid with 25% sucrose in TNE (150 mM NaCl, 10 mM Tris [pH 8.0], 2 mM EDTA [pH 8.0]) and then centrifuged at 78,000 × *g* for 2 h at 4°C. The supernatants were disposed of and the remaining virion pellets were resuspended in TNE. Stocks of KSHV-BAC16 were titrated by first infecting iLEC with serially diluted virus preparations. After 48 h, the cells were harvested and evaluated for GFP expression via flow cytometry. All *de novo* infections were conducted using an MOI that results in 80% GFP-expressing iLEC 48 h after infection.

### Antibodies and Other Reagents

A rabbit anti-KSHV ORF65 antibody (generously provided by Dr. Bala Chandran, Rosalind Franklin University of Medicine and Science, Chicago, IL, USA) was diluted 1:1000 and used to detect the viral capsid by immunofluorescence (IFA) in combination with an Alexa Fluor 488-conjugated anti-rabbit secondary antibody (1:1000, Invitrogen, Eugene, OR, USA). Rhodamine phalloidin (Invitrogen, Eugene, OR, USA) was diluted 1:40 to detect actin filaments via IFA. An anti-HO-1 antibody (BD Transduction Laboratories, San Jose, CA, USA) and an anti-β-actin-HRP conjugated antibody (Sigma, St. Louis, MO, USA) were used in western blot procedures at a dilution of 1:1,000 and 1:20,000, respectively. An anti-mouse-HRP conjugated antibody (EMD Millipore, Billerica MA, USA) was used at a dilution of 1:10,000 as the secondary antibody for HO-1 detection. LPS at 100 ng/ml was used to induce TLR4 signaling. The TLR4 inhibitor CLI-095 (Invivogen) was used at a concentration of 3 μM. The HO-1 inhibitor OB-24 (Alaoui-Jamali et al., 2009) was kindly provided by Dr. Ajay Gupta (Osta Biotechnologies, Inc., Dollard-des-Ormeaux, QC, Canada) and was used at a concentration of 20 μM. The viral DNA polymerase inhibitor Foscarnet (FOS, Sigma) was used at a concentration of 400 μM. The CO releasing molecule CORM-2 (Sigma) was used at a concentration of 10 μM. Bilirubin (Sigma) was used at 20 μM. Ferric citrate (Sigma) was used at 20 μM. Bilirubin and ferric citrate were used in conjunction with CORM-2 in order to evaluate both their collective and individual contributions to TLR4-signaling in LPS-treated iLEC. The HO-1 substrate heme arginate (HA) was obtained from Orphan Europe (Nanterre, France) and used at 5 μM. When CORM-2 was used, DMSO was included as a vehicle control treatment. Where appropriate, PBS was used as a vehicle control for HA and OB-24, which are water-soluble compounds.

### Immunofluorescence and Image Analysis

For IFA experiments, cells were seeded onto collagen-coated coverslips (BD Biosystems, Franklin Lakes, NJ, USA) in six-well tissue culture plates. At the end of an experiment, the cells were fixed in 2% paraformaldehyde for 8 min, washed three times in phosphate-buffered saline (PBS), and permeabilized in 0.25% Triton X-100 for 8 min. Samples were next blocked [20% normal goat serum (NGS) in PBS] for 20 min at 37°C and then incubated with primary antibody in PBS with 1% NGS for 1 h at 37°C. Coverslips were then washed three times in PBS and incubated with a combination of the appropriate secondary antibody, rhodamine phalloidin for actin filament staining, and the nuclear stain 4,6-diamidino-2-phenylindole (DAPI) for 45 min at 37°C. Coverslips were then washed three times in PBS and mounted on glass slides with Fluoromount-G (Southern Biotech, Birmingham, AL, USA). Prepared slides were analyzed with a DeltaVision real-time deconvolution fluorescence microscope (Applied Precision, Issaquah, WA, USA), and images were captured with a Photometrics CoolSNAP HQ camera. Image analysis was performed with SoftWoRx (Applied Precision). Stacks of images (0.2-μm z-step) were captured at 60× magnification. Stacks were subjected to deconvolution, and two- or three-section projections were made by superimposing representative *z*-planes to generate the final images.

### Carbon Monoxide Sensor

A plasmid construct expressing the fluorescent CO sensor protein COSer was kindly provided by Dr. Chuan He (University of Chicago, Chicago, IL, USA). COSer is a cpVenus-CooA fusion protein that fluoresces in the presence of CO (Wang et al., 2012). The cpVenus moiety is a circularly permuted YFP variant, while CooA is a CO-sensing protein produced by *Rhodospirillum rubrum*. The gene encoding COSer was subcloned into the lentiviral vector pCDH-CMV-MCS-EF1-Puro (System Biosciences, Palo Alto, CA, USA) using standard methods. Lentiviral particles derived from the resulting pCDH-COSer construct were then used to generate a stable HeLa cell line expressing the CO sensor (HeLa-COSer). HeLa-COSer cells were then used as a tractable system with which to measure endogenous CO in response to specific stimuli. Flow cytometry for cpVenus fluorescence was then used to assess intracellular CO levels in HeLa-COSer cells subjected to various experimental conditions.

### Reverse Transcription-qPCR

Extraction of total RNA was performed using RNeasy minikits (Qiagen, Valencia, CA, USA). A DNase I (Qiagen) digestion step was included to eliminate any residual DNA. Total cDNA was generated from these samples with the SuperScript III First-Strand Synthesis System (Invitrogen; Life Technologies, Carlsbad, CA, USA). The following gene-specific primers were used for qPCR assays: GAPDH F, 5′-GAAGGTGAAGGTCGGAGT-3′; GAPDH R, 5′-GAAGATGGTGATGGGATTTC-3′; HO-1 F, 5′-GCCCTTCAGCATCCTCAGTTC-3′; HO-1 R, 5′-GGTTTGAGACAGCTGCCACA-3′; ORF59 F 5′-CGAGTCTTCGCAAAAGGTTC-3′; ORF59 R, 5′-AAGG GACCAACTGGTGTGAG-3′; IL-1β F 5′-GCCAATCTTCATTGCTCAAGTGT-3′, IL-1β R 5′-AGCCATCATTTCACTGGCGA-3′; LANA-1 F 5′- CGCGAATACCGCTATGTAC-3′, LANA1 R 5′-CTGGAAGGCCTGAGATAA-3′; K5 F 5′-ACAAGGACCGTCAATTCGATG-3′, K5 R 5′-TGCCATACCGACGGCC-3′; IL-6 F 5′- CCAGGAGCCCAGCTATGAAC-3′, IL-6 R 5′-CCCAGGGAGAAGGCAACTG-3′; IFNβ F 5′-ACGCCGCATTGACCATCTA-3′, IFNβ R 5′-TAGTCTCATTCCAGCCAGTGC-3′. Nuclear and cytoplasmic fractionations were performed using the Nuclei EZ Prep Kit (Sigma), and the resulting fractions were then subjected to DNA extraction using the Blood and Tissue DNA kit (Qiagen). For detection of genomic targets, the following primers were used: ORF26 F 5′-ACGCGAAAGGATTCCACCAT-3′, ORF26 R 5′-TCCGTGTTGTCTACGTCCA-3′; GAPDH F 5′-TGCCTTCTTGCCTCTTGTCTCT-3′, GAPDH R 5′-GGCTCACCATGTAGCACTACC-3′ (nuclear marker); MT-ND1 F5′-CGGAGTAATCCAGGTCGGTT-3′ MT-ND1 R 5′-AGGCGCTTTGTGAAGTAGGC-3′ (cytoplasmic marker). For qPCR assays, 5 μl of cDNA or DNA (typically 10 ng) was mixed with gene specific primers plus 12.5 μl of Power SYBR Green PCR Master Mix (ABI, Foster City, CA, USA) in a final volume of 25 μl. Optimization of primer and sample concentrations was performed for all targets. For qPCR assays, standard curves were established using appropriate control samples and a dilution series ranging from 100 ng to 3.2 pg of cDNA/DNA. Sample amplification was performed in an ABI real-time PCR 7500 system. For relative quantitation experiments, the signal for each cDNA was normalized to GAPDH cDNA levels. KSHV genome copy numbers were normalized to host genome copies (GAPDH) and are displayed as either viral genome copies per 100 ng of DNA or viral genomes per host genome at 2 h post-infection.

### Flow Cytometric Analysis

Cells to be analyzed via flow cytometry were first dissociated from tissue culture plates using non-enzymatic Cellstripper (25-056-CI; CellGro/Corning, Manassas, VA, USA) and then pelleted at 1800 × *g* at 4°C for 5 min. The cells were then fixed using 2% PFA, incubated on ice for 15 min, pelleted, washed twice in 1X Washing Buffer (1X PBS, 2% NGS, 0.1% sodium azide), resuspended in 1X Washing Buffer and finally analyzed on an LSR2 Flow Cytometer (BD Biosciences, San Diego, CA, USA).

### Western Blotting

Cells were lysed in radio immunoprecipitation assay buffer (50 mm Tris [pH 7.5], 150 mm NaCl, 1% Nonidet P-40, 0.5% deoxycholate, 0.1% SDS, 1x Complete Protease Inhibitor [Roche Applied Science, Pleasanton, CA, USA] and Phosphatase inhibitors), incubated on ice for 30 min, and then centrifuged at 13,400 × *g* for 10 min at 4°C. Cleared supernatants were transferred to fresh tubes, and protein concentrations were determined via a bicinchoninic acid (BCA) assay (Thermo Fisher Scientific, Waltham, MA, USA). Proteins were separated via reducing SDS-PAGE, transferred to polyvinylidene difluoride membranes, incubated in blocking buffer (5% Bovine Serum Albumin in Tris-buffered saline with 0.1% Tween 20 [TBST]) for 1 h, and then probed with the appropriate primary antibody diluted in blocking buffer. Blots were then washed three times with TBST, incubated with the appropriate secondary antibodies in blocking buffer, and then washed a further three times with TBST. Blots were developed with ECL western blotting substrate (Thermo Fisher Scientific), and the resulting data were captured with G:BOX (Syngene, Frederick, MD, USA). The images shown are representative of two independent experiments.

### Bilirubin ELISA

Intracellular bilirubin levels were detected using a human total bilirubin (TBIL) ELISA Kit (MyBiosource, San Diego, CA, USA). The TBIL ELISA is compatible for use with cell lysates and, by measuring the heme metabolite bilirubin, provides a functionally equivalent readout of HO-1 enzyme activity. Procedures followed were as described in the manufacturer’s manual. Briefly, standards and test samples were incubated with anti-bilirubin HRP-conjugated antibody in pre-coated plates for 1 h. Wells were then washed three times and incubated with the substrate. After addition of a stop solution, plates were read at 450 nm in a microplate reader.

### Statistical Analysis

Quantitative PCR data is expressed as the mean ± Standard Error of the Mean (SEM). One-way ANOVA was used to compare means from different groups.

## Results

### KSHV Infection Enhances the Production of Intracellular CO to Reduce Production of TLR-4-Activated Antiviral Cytokines

**FIGURE 1:**
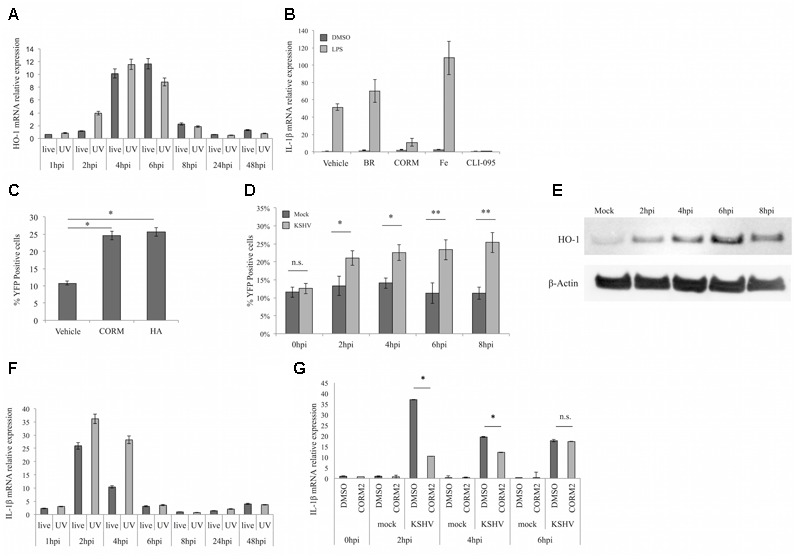
**Kaposi sarcoma herpesvirus (KSHV) infected endothelial cells produce carbon monoxide (CO) which reduces production of TLR4-derived cytokines. (A)** qPCR for heme oxygenase-1 (HO-1) message in iLEC infected with live KSHV-BAC16 (live, dark gray) or UV-inactivated KSHV-BAC16 (UV, light gray) at different times post-infection. Data are expressed as the mean ± SEM (*n* = 3). **(B)** qPCR for IL-1β message in iLEC pretreated for 2 h with DMSO (Vehicle), 20 μM Bilirubin (BR), 20 μM Ferric citrate (Fe), or 3 μM TLR4 inhibitor (CLI-095) or for 10 min with 10 μM CORM-2 and then stimulated with 100 ng/ml LPS for 4 h (light gray) or DMSO (dark gray) as a vehicle control. Data are expressed as the mean ± SEM (*n* = 3). **(C)** Flow cytometric analysis of HeLa-COSer cells treated with DMSO control (vehicle), 10 μM CORM-2 or 5 μM heme arginate (HA). Shown is the percentage of YFP positive cells at the time of peak CO production; 20 min for CORM-2 and 4 h for HA or vehicle. Data are expressed as the mean ± SEM (*n* = 3). **(D)** Flow cytometric analysis of HeLa-COSer cells that were mock-infected (dark gray) or KSHV-infected (light gray) and collected at early times (0–8 hpi) post-infection. CO production is measured by quantitation of the percentage of YFP positive cells. Data are expressed as the mean ± SEM (*n* = 3). **(E)** Western blot for HO-1 protein expression in HeLa-COSer cells infected with KSHV for 2, 4, 6, and 8 h. β-actin was used as the loading control. Data are representative of two independent experiments (*n* = 2). **(F)** qPCR for IL-1β message in iLEC infected with live KSHV-BAC16 (live, dark gray) or UV-inactivated KSHV-BAC16 (UV, light gray) at different times post-infection. Data are expressed as the mean ± SEM (*n* = 3). **(G)** qPCR for IL-1β message in iLEC pretreated with DMSO control (dark gray) or 10 μM CORM-2 (light gray) for 10 min and then infected with KSHV-BAC16. Data are expressed as the mean ± SEM (*n* = 3). ^∗^*p*-value ≤ 0.05, ^∗∗^*p*-value ≤ 0.01. A one-way ANOVA test was used for statistical analysis.

We previously demonstrated that *de novo* infection of iLEC with KSHV induces expression of the host enzyme HO-1 with an early, transient phase followed by a sustained phase associated with the establishment of latency (Botto et al., 2015). In the current study, we sought to investigate if the early phase of HO-1 induction plays a role in KSHV infection. We began by validating our prior observations (Botto et al., 2015), reconfirming that KSHV-infected iLEC exhibit a transient peak of HO-1 message between 4 and 6 h post-infection (hpi) and that this early HO-1 induction is observed regardless of whether the viral inoculum is live- or UV-inactivated (**Figure 1A**).

Since HO-1 catabolizes heme into CO, free iron, and biliverdin, which is then rapidly reduced to bilirubin by biliverdin reductase, we considered the possibility that any or all of these biologically active metabolites could influence the viral infection process. We were particularly interested in the gasotransmitter CO, as CO has been shown to inhibit activation of LPS-induced TLR4 (Wang et al., 2009) while TLR4 has been identified as a pathogen sensor mediating innate immunity to KSHV (Lagos et al., 2008). To evaluate the influence of HO-1 metabolites on TLR4 activation, iLEC were pretreated with DMSO vehicle, bilirubin (BR), CO-releasing molecule (CORM-2), Ferric citrate (Fe) or the TLR4 inhibitor CLI-095 for the times indicated and then stimulated with LPS or DMSO alone for another 4 h. Expression of the inflammatory cytokines IL-1β (**Figure 1B**) and IFNβ (Supplementary Figure S1) was then measured by qPCR as a readout of TLR4 activation. This experiment revealed that CO was the only heme metabolite able to block TLR4-signaling in LPS-treated iLEC. Specifically, iLEC treated with exogenous CO produced significantly reduced levels of IL-1β and IFNβ in response to LPS stimulation relative to cells treated with vehicle alone. The ability of the TLR4 inhibitor CLI-095 to similarly block cytokine production in response to LPS stimulation supported the TLR4-specificity of CO-mediated inhibition. Conversely, iLEC treated with the other heme metabolites (Fe and to a lesser extent BR) remained responsive to TLR4 activation. These experiments also verified that HO-1 message levels were not significantly altered by LPS stimulation, but were moderately induced by CORM-2, the latter likely reflecting the cytoprotective potential of CO (Supplementary Figure S2).

The above experiments confirmed that exogenous CO (via CORM-2) can inhibit TLR4 signaling. We next asked whether KSHV-induced HO-1 was able to raise endogenous CO to similar levels. In order to detect endogenous CO, we employed the fluorescent CO sensor COSer (Wang et al., 2012). We first generated a stable HeLa cell line (HeLa-COSer) expressing COSer and tested it for induction of fluorescence in the presence of either CORM-2 or heme arginate (HA), the latter of which is both an inducer and a substrate for HO-1. As expected, HeLa-COSer cells exposed to either CORM-2 or HA showed a significant increase in the percentage of YFP positive cells compared to DMSO-treated controls (**Figure 1C**). The time difference for peak of CO production (20 min for CORM-2 and 4 h for HA) likely reflects the fact that CORM-2 functions to deliver exogenous CO, while endogenous CO produced as a downstream metabolite of HA requires HO-1 enzyme activity. Having validated the HeLa-COSer system as a means to measure temporal increases in endogenous CO, we next evaluated the effect of KSHV infection on endogenous CO levels over the intervals post-infection that spanned the duration of KSHV-triggered early HO-1 induction (**Figure 1D**). Importantly, YFP fluorescence was significantly enhanced in HeLA-COSer cells infected with KSHV, at a time coincident with the kinetics of early HO-1 induction observed in KSHV-infected iLEC (**Figure 1E**). Taken together, these data suggest that early KSHV induction of HO-1 is indeed sufficient to raise levels of intracellular CO.

Having demonstrated (**Figure 1B** and Supplementary Figure S1) that IL-1β and IFNβ expression can be used to measure LPS-dependent TLR4 signaling, we next used these parameters to test the KSHV-dependent induction of TLR4 signaling at the times co-incident with early HO-1 induction. As shown in **Figure 1F**, levels of IL-1β message in cells infected with both live and UV-inactivated virus were increased as early as 2 hpi (**Figure 1F**). By 4 hpi, IL-1β transcription was sustained in cells infected with UV-inactivated KSHV, but decreased in cells infected with live virus, likely due to *de novo* expression of viral immune evasion proteins. Because CO is able to inhibit TLR4 signaling (**Figure 1B**) and because KSHV infection activates TLR4 signaling (Lagos et al., 2008), we next tested whether the expression of IL-1β and IFNβ in KSHV-infected iLEC could be reduced by delivering CO to iLEC immediately prior to infection. Indeed, iLEC pretreated with CORM-2 and then infected with KSHV showed significantly lower levels of IL-1β compared to control cells [**Figure 1G**; similar trends were observed for IFNβ (dns)], indicating that CO is able to block TLR4 signaling and reduce the production of antiviral cytokines in response to KSHV infection.

### TLR4 Inhibition via CO or a TLR4 Inhibitor Facilitates KSHV Infection of iLEC

**FIGURE 2:**
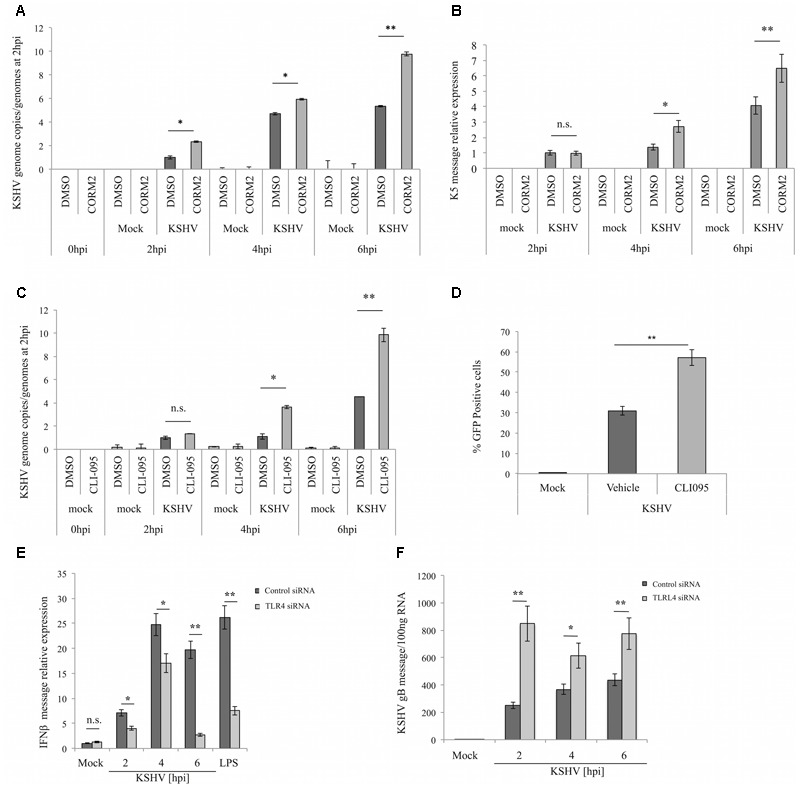
**TLR4 inhibition via CO or TLR4 inhibitor CLI-095 facilitates KSHV infection of iLEC. (A)** qPCR for KSHV genome copies in iLEC pretreated with DMSO control (dark gray) or 10 μM CORM-2 (light gray) for 10 min and then infected with KSHV-BAC16. KSHV genome copies are represented as relative to the number of genomes present at 2 hpi. Data are expressed as the mean ± SEM (*n* = 3). **(B)** qPCR for KSHV K5 transcript in iLEC pretreated with DMSO control (dark gray) or 10 μM CORM-2 (light gray) for 10 min and then infected with KSHV-BAC16. KSHV K5 levels are represented as relative to K5 levels measured at 2 hpi. Data are expressed as the mean ± SEM (*n* = 3). **(C)** qPCR for KSHV genome copies in iLEC pretreated with DMSO control (dark gray) or 3 μM CLI-095 (light gray) and then infected with KSHV-BAC16. KSHV genome copies are represented as relative to the number of genomes present at 2 hpi. Data are expressed as the mean ± SEM (*n* = 3). **(D)** Flow cytometry for iLEC pretreated with DMSO (Vehicle) or 3 μM TLR4-inhibitor (CLI-095) and then infected with KSHV-BAC16. Infection is quantified by determining the percentage of GFP positive cells present at 24 hpi. Data are expressed as the mean ± SEM (*n* = 3). qPCR for IFNβ **(E)** and gB **(F)** message levels in iLEC transfected overnight with Control siRNA (dark gray) or TLR4 siRNA (light gray) and then infected with KSHV for 2, 4, and 6 h. LPS (100 ng/ml for 4 h) was used as a control stimulus for TLR4 activation. Data are expressed as the mean ± SEM (*n* = 3). A one-way ANOVA test was used for statistical analysis. ^∗^*p*-value ≤ 0.05, ^∗∗^*p*-value ≤ 0.01.

Innate immune activation in response to viral challenge is important for the induction of interferon-stimulated genes (ISGs), the products of which target all steps of the infection (Schoggins and Rice, 2011). Since CO treatment of iLEC led to a reduction in the KSHV-dependent production of the antiviral cytokines IL-1β and IFNβ, we next determined whether this same treatment had any impact on the ability of KSHV to infect iLEC. Indeed raising CO levels via CORM-2 treatment of cells prior to KSHV infection resulted in a modest but significant increase in the number of viral genomes (**Figure 2A**) and the early viral transcript K5 (**Figure 2B**) detected at early times (2, 4, and 6 h) post-infection. As expected, a similar phenotype was observed in iLEC treated with the TLR4 inhibitor CLI-095 prior to KSHV infection (**Figure 2C**). To confirm that TLR4 inhibition does indeed facilitate KSHV infection, iLEC pretreated with CLI-095 were evaluated for GFP expression via flow cytometry 24 h after infection with KSHV-BAC16. Again, pretreatment of iLEC with the TLR4 inhibitor resulted in detection of a significantly higher percentage of KSHV-infected (GFP positive) cells as compared to cells treated with a vehicle (DMSO) control (**Figure 2D**). Further support for this notion was provided via siRNA-dependent knockdown of TLR expression, which resulted in the reduced expression of the antiviral cytokine IFNβ as well as the enhanced expression of the KSHV lytic marker gB (**Figures 2E,F**). Note that CLI-095 treatment of iLEC did not significantly impact HO-1 expression, indicating that any effect observed with this inhibitor was attributable to its effect on TLR4, and not to non-specific modulation of HO-1 (Supplementary Figure S3). Together, these data suggest that TLR4 inhibition, whether achieved through CO treatment or chemical inhibition, renders iLEC more permissive to KSHV infection.

### Inhibition of HO-1 Activity in iLEC Results in Decreased CO Production and Reduced KSHV Infection

**FIGURE 3:**
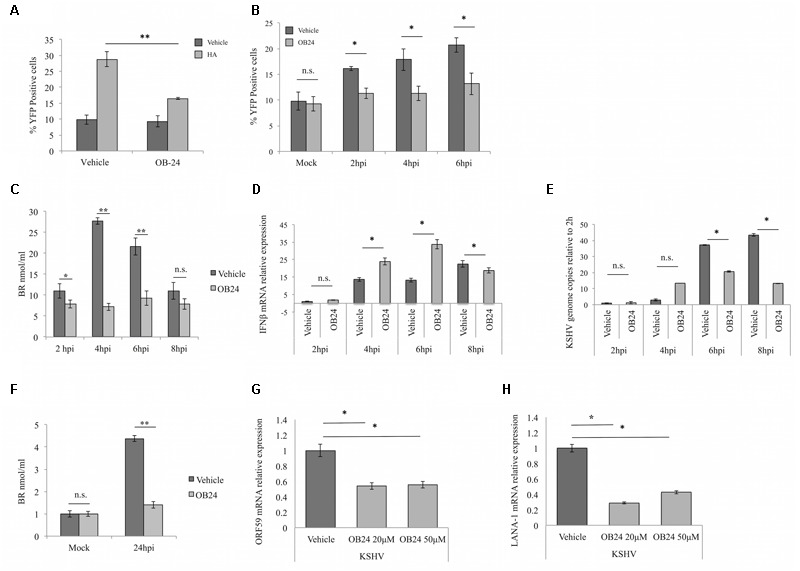
**Heme oxygenase-1 inhibition results in decreased KSHV infection in iLEC and CO production. (A)** Flow cytometric analysis of HeLa-COSer cells pretreated overnight with PBS control (Vehicle) or 20 μM HO-1 inhibitor OB-24 (OB-24) and then stimulated with PBS (Vehicle; dark gray) or 5 μM heme arginate (HA; light gray) for 4 h. CO production is measured by percentage of YFP positive cells. Data are expressed as the mean ± SEM (*n* = 3). **(B)** Flow cytometry for HeLa-COSer cells pretreated with PBS vehicle (dark gray) or 20 μM of the HO-1 enzyme activity inhibitor OB-24 (light gray) overnight and then infected with KSHV. CO production is measured by quantitation of the percentage of YFP positive cells. Data are expressed as the mean ± SEM (*n* = 3). **(C)** ELISA for intracellular bilirubin levels as a measure of HO-1 activity. iLEC were pre-treated with PBS (Vehicle; dark gray) or 20 μM OB-24 (light gray) overnight, infected with KSHV-BAC16 and then harvested for analysis at different times post-infection (2, 4, 6, and 8 hpi). Data are expressed as the mean ± SEM (*n* = 2). **(D,E)** qPCR for IL-1β mRNA **(D)** and KSHV genome copies **(E)** in iLEC pretreated overnight with PBS (Vehicle, dark gray) or 20 μM OB-24 (OB24; light gray), infected with KSHV-BAC16 and harvested for analysis at 2, 4, 6, and 8 hpi. Data are expressed as the mean ± SEM (*n* = 3). KSHV genome copies are represented as relative to the number of genomes present at 2 hpi. **(F)** ELISA for intracellular bilirubin levels in iLEC pretreated with PBS (Vehicle; dark gray) or 20 μM OB-24 (light gray) overnight and then infected with KSHV for 24 h before harvest and analysis. Data are expressed as the mean ± SEM (*n* = 2). **(G,H)** qPCR for KSHV lytic transcript ORF59 **(G)** and latent transcript LANA-1 **(H)** in iLEC pretreated overnight with PBS (Vehicle; dark gray) or with OB-24 at 20 μM or 50 μM (light gray) and then infected with KSHV-BAC16 for 24 h before harvest and analysis. KSHV transcript levels are represented as relative to transcript levels measured at 2 hpi. Data are expressed as the mean ± SEM (*n* = 3). ^∗^*p*-value ≤ 0.05, ^∗∗^*p*-value ≤ 0.01. A one-way ANOVA test was used for statistical analysis.

As shown above, KSHV infection of HeLaCOSer cells leads to both HO-1 induction (**Figure 1E**) and CO generation (**Figure 1D**) at early times post-infection. Our next goal was to directly link CO production with HO-1 activity. For this purpose, we employed the HO-1 inhibitor OB-24, an imidazole-based water-soluble small-molecule inhibitor that has demonstrated potent anti-tumor and anti-metastatic activity in preclinical tumor models (Alaoui-Jamali et al., 2009). Contrary to other commonly used HO-1 inhibitors, OB-24 selectively inhibits HO-1 enzyme activity and does not influence HO-1 protein expression (Kinobe et al., 2008). For the first of these experiments, HeLa-COSer cells were pretreated overnight with OB-24 or vehicle and then exposed to the HO-1 inducer and substrate HA. As expected, HA treatment alone led to a significant increase in CO production as measured by YFP fluorescence, while pre-treatment of the cells with OB-24 largely blocked this production, demonstrating the correlation between HO-1 activity and CO production (**Figure 3A**). Next, HeLa-COSer cells pretreated overnight with OB-24 or PBS vehicle were infected with KSHV, harvested at early intervals (2–6 hpi) and analyzed for YFP fluorescence. Importantly, YFP expression was significantly reduced in KSHV-infected HeLa-COSer cells pretreated with OB-24 as compared to vehicle-treated cells (**Figure 3B**). These data demonstrate that KSHV-induced HO-1 does indeed lead to elevation of endogenous CO.

We next performed similar experiments in endothelial cells. We initially established a non-toxic but effective dose-range for OB-24 by staining iLEC for the apoptotic markers Annexin V and propidium iodide (Supplementary Figure S4). With this value established, OB-24 was then tested for its capacity to inhibit HO-1 activity in KSHV-infected iLEC, as well as its potential to affect the host antiviral response and impact KSHV infection. Accordingly, iLEC were pretreated overnight with Vehicle (PBS) or OB-24. The next day, the cells were infected with KSHV, harvested at intervals spanning the early HO-1 induction peak (2–8 hpi) and then evaluated for bilirubin generation (Rücker and Amslinger, 2015) (**Figure 3C**), IFNβ expression (**Figure 3D**) and viral genome production (**Figure 3E**). As expected, treatment of iLEC with OB-24 abolished the peak of HO-1 activity that occurs following *de novo* KSHV infection, and this reduced activity correlated with both increased levels of IFNβ transcripts and reduced viral genome copy numbers. When iLEC pretreated with OB-24 and infected with KSHV for 24 hpi were analyzed, inhibition of HO-1 activity was maintained (**Figure 3F**) and cells expressed significantly lower levels of representative lytic (ORF59; **Figure 3G**) and latent (LANA-1; **Figure 3H**) viral transcripts. Thus, the data obtained using chemical inhibition of HO-1 activity further support the hypothesis that inhibition of HO-1 activity in iLEC increases the potency of the antiviral response and reduces the efficiency of *de novo* KSHV infection.

### TLR4 Inhibition Influences a Step in the Infection Process Subsequent to Virus Binding, Entry and Genome Delivery to the Nucleus

**FIGURE 4:**
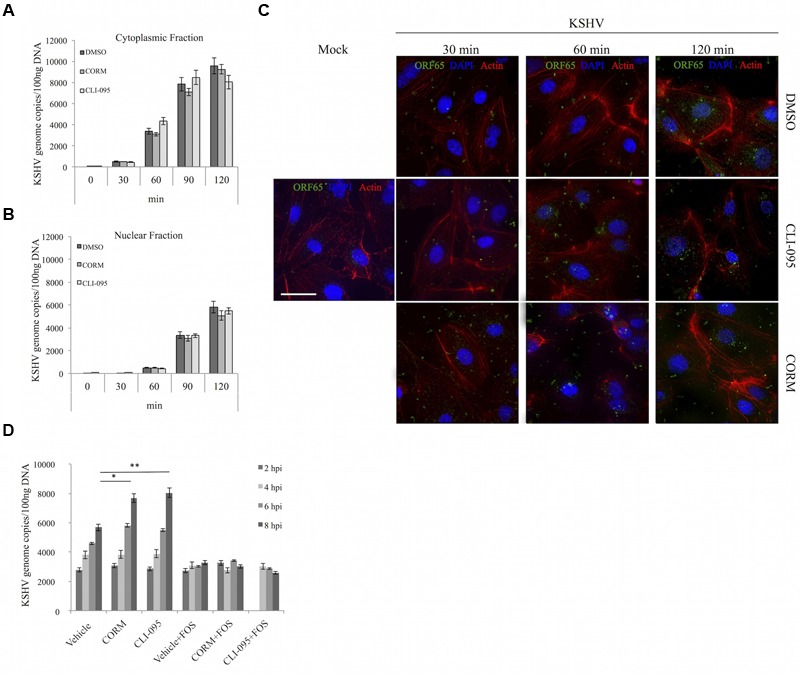
**TLR4 inhibition via CO or CLI-095 does not affect virus binding, entry or genome delivery to the nucleus.** qPCR for KSHV genome copies in **(A)** the cytoplasmic fraction and **(B)** the nuclear fraction of iLEC pretreated with 3 μM CLI-095 (pale gray) for 2 h or 10 μM CORM-2 (light gray) for 10 min and then infected with KSHV-BAC16. DMSO (dark gray) was used as a vehicle control. KSHV genome copies are represented as number of genomes per 100 ng DNA. Data are expressed as the mean ± SEM (*n* = 3). **(C)** Immunofluorescent analysis of iLEC infected with KSHV-BAC16 for 30, 60, or 120 min. Expression of ORF65 (green), Actin (red), and DAPI (blue) are shown. Single left panel: Uninfected cells (mock). Top three panels: DMSO-treated iLEC. Middle three panels: CLI-095 treated iLEC. Bottom three panels: CORM-2-treated iLEC. Images were captured at 60× magnification (scale bar = 30 μm). **(D)** qPCR for KSHV genome copies in iLEC pretreated with 3 μM CLI-095 for 2 h or 10 μM CORM-2 for 10 min and then infected with KSHV-BAC16 for up to 8 h in the presence or absence of 400 μM Foscarnet (+FOS). DMSO was used as a vehicle control. KSHV genome copies (2–8 hpi) are represented as number of genomes per 100 ng DNA. Data are expressed as the mean ± SEM (*n* = 3). A one-way ANOVA test was used for statistical analysis. ^∗^*p*-value ≤ 0.05, ^∗∗^*p*-value ≤ 0.01.

As illustrated in **Figures 1, 2**, TLR4 inhibition with either CLI-095 or CORM-2 renders iLEC more permissive to KSHV infection. To determine which step of the *de novo* infection process was affected, the various stages of the KSHV infection process were evaluated. iLEC were first treated with a vehicle control (DMSO), CORM-2 or CLI-095 for the indicated times, then infected with KSHV at 4°C, in order to permit virus binding but not entry into the host cell. After 30 min at 4°C, cells were either collected to determine the number of bound virions or further incubated at 37°C for 60, 90, or 120 min to allow for virus entry and nuclear trafficking. In order to ensure detection of only intracellular virus at these later time points, surface-bound virions were removed by washing the cells with citrate buffer (pH = 3.0) prior to collection. In order to assess viral genome delivery to the nucleus, infected iLEC were subjected to nuclear-cytoplasmic fractionation, and DNA was then extracted from the two fractions. Subsequent quantitation of KSHV genome copies via qPCR showed that neither CO nor CLI-095 treatment impacted the number of viral genomes able to enter the cell (**Figure 4A**) or reach the nucleus (**Figure 4B**). In a parallel approach to confirm this result, KSHV entry was assessed via immunofluorescence detection of the viral capsid protein ORF65. Actin-phalloidin and DAPI staining were included in order to visualize the subcellular localization of the viral particles. Regardless of the treatment utilized, there was no significant difference in the number of capsids observed at 30 min (binding), 60 min (entry), or 120 min (transport to the nucleus) post-infection (**Figure 4C**). Collectively, the data from these experiments suggests that neither chemical nor CO-based inhibition of TLR4 signaling has a measurable impact on the initial stages of KSHV infection of iLEC that culminate in nuclear delivery of the viral genome. To further substantiate this result, we next employed the viral polymerase inhibitor foscarnet (FOS) to evaluate intracellular levels of viral DNA in the absence of viral replication, thus allowing for measurement of only input viral genomes. iLEC were pretreated for 2 h with CLI-095 or for 10 min with CORM-2 and then treated with FOS for 1 h prior to infection. The cells were subsequently infected with KSHV, collected at intervals from 2 to 8 hpi and analyzed via qPCR for viral DNA content (**Figure 4D**). As expected, treatment with FOS blocked viral DNA replication. Importantly, in the presence of FOS, similar amounts of viral DNA were present at 2 hpi in CO, CLI-095 and vehicle-treated cells. In agreement with results shown in **Figures 2, 3**, CO and CLI-095 treatment in the absence of FOS increased the number of intracellular viral genomes detected at later time points (6 and 8 hpi). Taken together, our data suggest that inhibition of TLR4 activation benefits a phase of KSHV infection that occurs following viral genome delivery to the nucleus.

## Discussion

Kaposi sarcoma herpesvirus infection of endothelial cells results in robust expression of the inducible isoform of heme oxygenase, HO-1, both *in vivo* and *in vitro* (McAllister et al., 2004; Botto et al., 2015). *In vitro*, HO-1 appears to be upregulated in a biphasic manner by two distinct mechanisms. The first of these is operational at early times after *de novo* infection, and the second comes into play once the virus has established latency (Botto et al., 2015). In the present study we investigated the consequences of HO-1 induction during the early stages of *de novo* KSHV infection. KSHV infection of target cells is known to trigger activation of host signaling pathways that promote viral entry and delivery of viral DNA to the nucleus (Chandran, 2010). At the same time, the infection process activates a variety of host innate immune responses, including the TLR4 signaling pathway, to induce an antiviral state (Lagos et al., 2008). KSHV encodes several proteins and microRNAs that have been shown to subvert the antiviral innate immune response. In many instances, the host proteins targeted by particular viral factors have been identified, and this is particularly true in the context of innate immune evasion mechanisms adopted by KSHV to evade or manipulate the IFN response (Sathish and Yuan, 2011; Brulois and Jung, 2014; Lee et al., 2015). While the specific viral factor(s) that transiently upregulates HO-1 to facilitate CO-mediated suppression of the TLR4 pathway remains to be identified, our data predict that a virion component is responsible, thereby allowing the inhibition of innate host defenses immediately upon infection. This method of HO-1 induction is both distinct from and independent of the KSHV-dependent induction of HO-1 during latency, for which the mechanism has been previously defined (Gjyshi et al., 2014; Botto et al., 2015). This separation suggests that HO-1 may play temporally and functionally distinct roles during the KSHV life cycle, first by facilitating the initial infection event and later participating in the successful establishment of viral latency.

In this report, we describe a novel mechanism of TLR4 inhibition in KSHV-infected LEC that is mediated by HO-1 and CO, an endogenous gasotransmitter produced through the catabolism of heme. Shortly after *de novo* infection, KSHV activates both the host TLR4 signaling pathway and expression of HO-1, and then the CO generated as a result of KSHV-induced HO-1 activity downmodulates TLR4 signaling and promotes KSHV infection. We found that when KSHV-infected LEC are pretreated with either a TLR4 inhibitor or exogenous CO, they support increased viral gene expression and generate higher genome copy numbers. Moreover, cells subjected to elevated levels of CO express reduced levels of IL-1β and IFNβ, two antiviral cytokines associated with TLR4 activation. Importantly, in response to early HO-1 induction, KSHV-infected cells contain measurably higher levels of intracellular CO, indicating that HO-1 activity in infected cells can indeed increase endogenous CO levels. In support of this conclusion, cells pretreated with an inhibitor of HO-1 enzyme activity are less permissive to KSHV infection, produce higher levels of inflammatory cytokines and contain less CO. Collectively, these data suggest that HO-1-activity liberates CO from heme at levels sufficient to inhibit the TLR4-activated antiviral response to *de novo* KSHV infection.

We attempted to identify the stages of KSHV infection that are impacted by CO-mediated suppression of TLR4 signaling. TLR4 signaling is known to activate a series of pathways that culminate in the transcription of genes encoding inflammatory cytokines and chemokines, as well as type I IFN. IFNβ secretion from infected cells results in the activation of IFN-stimulated genes (ISGs) that initiate an antiviral program capable of acting on both infected cells and uninfected neighboring cells (Schoggins and Rice, 2011; Schneider et al., 2014). Certain antiviral ISGs, including MDA5, RIG-1, and TRIM proteins, are involved in the amplification and regulation of the IFN response (Schoggins and Rice, 2011). Others such as Mx, OAS, RNaseL, APOBEC3, and PKR are involved in antiviral mechanisms that interfere with the virus life cycle (Schoggins and Rice, 2011). Interestingly, we observed no significant difference in the number of KSHV virions able to bind, enter, or reach the nucleus of infected EC. In contrast, we detected significant differences in viral gene expression and genome copies at later times post-infection. One possible explanation is that CO-mediated inhibition of the TLR4-triggered antiviral response facilitates a phase of KSHV infection that occurs following viral genome delivery to the nucleus, potentially by impacting expression of ISGs acting on either the target and/or neighboring cells. Yet another possibility comes from recent studies that have identified host innate immune pathways initiated upon delivery of KSHV dsDNA genomes to the nucleus. These include activation of the DNA Damage Response (DDR) (Singh et al., 2014) as well as formation of nuclear inflammasomes, the latter of which is mediated by the interferon gamma-inducible protein 16 (IFI16) (Kerur et al., 2011). Interestingly, DDR signaling is important for successful establishment and maintenance of KSHV latency (Singh et al., 2014), and the same may be true for IFI16-mediated inflammasome induction. Alternately, if the IFI16 inflammasome represents an effective antiviral response, it is possible that KSHV has evolved a strategy to evade or usurp this mechanism. In this scenario, TLR4 represents another arm of the innate immune response that is induced during the early stages of KSHV infection, and KSHV usurps HO-1 to inhibit TLR4 signaling via a CO-mediated mechanism. Other studies have shown that KSHV binding to endothelial cells results in rapid activation of the TLR4 pathway (Lagos et al., 2008), and induction of NRF-2 (Gjyshi et al., 2014), the latter mechanism being essential for establishment of *de novo* infection. We can therefore envision a model whereby NRF-2 activation of HO-1 enzyme activity allows for production of the gasotransmitter CO, which in turn inhibits TLR4 signaling to facilitate establishment of KSHV infection after nuclear genome delivery. HO-1 homeostasis is then rapidly reestablished, likely via the action of the HO-1 repressor BACH-1. HO-1 is induced once again after the establishment of latency, due at least in part to KSHV miR-K12-11, which targets BACH-1 (Botto et al., 2015).

It is not unprecedented for herpesviruses to subvert host proteins to evade TLR4. For example, EBV induces the host microRNA miR-21 (Mrazek et al., 2007), which indirectly attenuates TLR4 signaling via the host protein PDCD4 (Sheedy et al., 2009). KSHV subversion of the TLR4 pathway has also been previously reported. For example, Lagos et al. (2008) demonstrated that ectopic expression of the viral lytic proteins vIRF1 and vGPCR leads to downregulation of both TLR4 mRNA and signal activation, while other groups have shown that the viral RTA decreases TLR4 protein levels (Bussey et al., 2014) and is able to further inhibit TLR signaling by degrading the TLR4/TLR3 adaptor TRIF (Ahmad et al., 2011). Given TLR4’s importance in innate immunity, it is not surprising that KSHV has evolved more than one way to limit the activation of this pathway and its downstream consequences. Interestingly, the mechanism we define herein is novel in that TLR4 signaling is attenuated indirectly by the virus via the action of yet another host protein, HO-1.

Heme oxygenase-1 and the heme metabolite CO have been shown to inhibit TLR4 signaling to reduce cytokine-driven inflammatory outcomes and protect cells against oxidative stress (Liu et al., 2002; Otterbein et al., 2005; Nakahira et al., 2006; Tsiftsoglou et al., 2006; Lin et al., 2009; Wang et al., 2009; Ruan et al., 2014; Xue and Habtezion, 2014). In addition to TLR4, CO also inhibits the activation of TLR2, TLR5, and TLR9 (Nakahira et al., 2006). However, among of this subset of TLRs, only TLR4 and TLR9, are directly activated by KSHV infection (Perry and Compton, 2006; Lagos et al., 2008). Whether the HO-1/CO axis acts as an endogenous inhibitor of TLR9 activation in the context of KSHV remains to be determined.

In addition to KS, HO-1 is highly induced in several other cancers, where it has been shown to contribute to tumor progression by promoting cell proliferation, angiogenesis, cytoprotection, and resistance to chemotherapy (Ryter and Tyrrell, 2000; Ryter et al., 2006; Lanceta et al., 2015). The current study, combined with our previous work demonstrating that KSHV-enhanced HO-1 activity promotes the growth of KSHV-infected EC, indicates that HO-1 can positively influence both the KSHV infection process and virus-driven tumorigenic mechanisms (McAllister et al., 2004). Here we show for the first time that HO-1 induction by KSHV facilitates viral infection by attenuating critical antiviral mechanisms. Identification of a dual role for HO-1 in promoting KSHV infection and tumorigenesis in endothelial cells further establishes its validity as a therapeutic target for KS.

## Author Contributions

Substantial contributions to the conception or design of the work: SB and AM. Acquisition and analysis of data for the work: SB and JG. Interpretation of data for the work: SB, JG, and AM. Drafting the work: SB. Revising the work critically for important intellectual content: JG and AM. Final approval of the version to be published: SB, JG, and AM. Agreement to be accountable for all aspects of the work in ensuring that questions related to the accuracy or integrity of any part of the work are appropriately investigated and resolved: SB, JG, and AM.

## Conflict of Interest Statement

The authors declare that the research was conducted in the absence of any commercial or financial relationships that could be construed as a potential conflict of interest.

## References

[B1] Ahmad H. Gubbels R. Ehlers E. Meyer F. Waterbury T. Lin R. (2011). Kaposi sarcoma-associated herpesvirus degrades cellular toll-interleukin-1 receptor domain-containing adaptor-inducing -interferon (TRIF). *J. Biol. Chem.* 286 7865–7872. 10.1074/jbc.M110.191452 21212282 PMC3048673

[B2] Alaoui-Jamali M. A. Bismar T. A. Gupta A. Szarek W. A. Su J. Song W. (2009). A novel experimental heme oxygenase-1-targeted therapy for hormone-refractory prostate cancer. *Cancer Res.* 69 8017–8024. 10.1158/0008-5472.CAN-09-0419 19808972

[B3] Boehme K. W. Compton T. (2004). Innate sensing of viruses by toll-like receptors. *J. Virol.* 78 7867–7873. 10.1128/JVI.78.15.7867-7873.2004 15254159 PMC446107

[B4] Botto S. Totonchy J. E. Gustin J. K. Moses A. V. (2015). Kaposi sarcoma herpesvirus induces HO-1 during *de novo* infection of endothelial cells via viral miRNA-dependent and -independent mechanisms. *mBio* 6:e00668 10.1128/mBio.00668-15 PMC4462627 26045540

[B5] Brulois K. Jung J. U. (2014). Interplay between Kaposi’s sarcoma-associated herpesvirus and the innate immune system. *Cytokine Growth Factor Rev.* 25 597–609. 10.1016/j.cytogfr.2014.06.001 25037686 PMC4252609

[B6] Brulois K. F. Chang H. Lee A. S.-Y. Ensser A. Wong L.-Y. Toth Z. (2012). Construction and manipulation of a new Kaposi’s sarcoma-associated herpesvirus bacterial artificial chromosome clone. *J. Virol.* 86 9708–9720. 10.1128/JVI.01019-12 22740391 PMC3446615

[B7] Bussey K. A. Reimer E. Todt H. Denker B. Gallo A. Konrad A. (2014). The gammaherpesviruses Kaposi’s sarcoma-associated herpesvirus and murine gammaherpesvirus 68 modulate the toll-like receptor-induced proinflammatory cytokine response. *J. Virol.* 88 9245–9259. 10.1128/JVI.00841-14 24899179 PMC4136288

[B8] Cesarman E. Mesri E. A. (2007). Kaposi sarcoma-associated herpesvirus and other viruses in human lymphomagenesis. *Curr. Top. Microbiol. Immunol.* 12 263–287. 10.1007/978-3-540-34344-8_10 17089801

[B9] Chandran B. (2010). Early events in Kaposi’s sarcoma-associated herpesvirus infection of target cells. *J. Virol.* 84 2188–2199. 10.1128/JVI.01334-09 19923183 PMC2820927

[B10] Chau L. Y. (2015). Heme oxygenase-1: emerging target of cancer therapy. *J. Biomed. Sci.* 22:22 10.1186/s12929-015-0128-0 PMC4380252 25885228

[B11] Chi P.-L. Chuang Y.-C. Chen Y.-W. Lin C.-C. Hsiao L.-D. Yang C.-M. (2014). The CO donor CORM-2 inhibits LPS-induced vascular cell adhesion molecule-1 expression and leukocyte adhesion in human rheumatoid synovial fibroblasts. *Br. J. Pharmacol.* 171 2993–3009. 10.1111/bph.12680 24628691 PMC4055201

[B12] Feng P. Moses A. Früh K. (2013). Evasion of adaptive and innate immune response mechanisms by γ-herpesviruses. *Curr. Opin. Virol.* 3 285–295. 10.1016/j.coviro.2013.05.011 23735334 PMC4397892

[B13] Gjyshi O. Bottero V. Veettil M. V. Dutta S. Singh V. V. Chikoti L. (2014). Kaposi’s sarcoma-associated herpesvirus induces Nrf2 during *de novo* infection of endothelial cells to create a microenvironment conducive to infection. *PLoS Pathog.* 10:e1004460 10.1371/journal.ppat.1004460 PMC4207826 25340789

[B14] Kerur N. Veettil M. V. Sharma-Walia N. Bottero V. Sadagopan S. Otageri P. (2011). IFI16 acts as a nuclear pathogen sensor to induce the inflammasome in response to Kaposi sarcoma-associated herpesvirus infection. *Cell Host Microbe* 9 363–375. 10.1016/j.chom.2011.04.008 21575908 PMC3113467

[B15] Kinobe R. T. Dercho R. A. Nakatsu K. (2008). Inhibitors of the heme oxygenase – carbon monoxide system: on the doorstep of the clinic? *Can. J. Physiol. Pharmacol.* 86 577–599. 10.1139/y08-066 18758507

[B16] Lagos D. Vart R. J. Gratrix F. Westrop S. J. Emuss V. Wong P.-P. (2008). Toll-like receptor 4 mediates innate immunity to Kaposi sarcoma herpesvirus. *Cell Host Microbe* 4 470–483. 10.1016/j.chom.2008.09.012 18996347 PMC2698447

[B17] Lanceta L. Mattingly J. M. Li C. Eaton J. W. (2015). How heme oxygenase-1 prevents heme-induced cell death. *PLoS ONE* 10:e134144 10.1371/journal.pone.0134144 PMC4535878 26270345

[B18] Lee H.-R. Amatya R. Jung J. U. (2015). Multi-step regulation of innate immune signaling by Kaposi’s sarcoma-associated herpesvirus. *Virus Res.* 19 1–13. 10.1016/j.virusres.2015.03.004 PMC4575611 25796211

[B19] Lin H. H. Chen Y. H. Yet S. F. Chau L. Y. (2009). After vascular injury, heme oxygenase-1/carbon monoxide enhances re-endothelialization via promoting mobilization of circulating endothelial progenitor cells. *J. Thromb. Haemost.* 7 1401–1408. 10.1111/j.1538-7836.2009.03478.x 19426286

[B20] Liu X.-M. Chapman G. B. Peyton K. J. Schafer A. I. Durante W. (2002). Carbon monoxide inhibits apoptosis in vascular smooth muscle cells. *Cardiovasc. Res.* 55 396–405. 10.1016/S0008-6363(02)00410-8 12123779

[B21] McAllister S. C. Hansen S. G. Ruhl R. A. Raggo C. M. DeFilippis V. R. Greenspan D. (2004). Kaposi sarcoma-associated herpesvirus (KSHV) induces heme oxygenase-1 expression and activity in KSHV-infected endothelial cells. *Blood* 103 3465–3473. 10.1182/blood-2003-08-2781 14726403

[B22] McAllister S. C. Moses A. V. (2007). Endothelial cell- and lymphocyte-based in vitro systems for understanding KSHV biology. *Curr. Top. Microbiol. Immunol.* 312 211–244. 10.1007/978-3-540-34344-8_8 17089799

[B23] Moore P. S. Chang Y. (1998). Kaposi’s sarcoma-associated herpesvirus-encoded oncogenes and oncogenesis. *J. Natl. Cancer Inst. Monogr.* 1998 65–71. 10.1093/oxfordjournals.jncimonographs.a024176 9709306

[B24] Moses A. V. Fish K. N. Ruhl R. Smith P. P. Strussenberg J. G. Zhu L. (1999). Long-term infection and transformation of dermal microvascular endothelial cells by human herpesvirus 8. *J. Virol.* 73 6892–6902. 10400787 10.1128/jvi.73.8.6892-6902.1999 PMC112774

[B25] Mrazek J. Kreutmayer S. B. Grasser F. A. Polacek N. Huttenhofer A. (2007). Subtractive hybridization identifies novel differentially expressed ncRNA species in EBV-infected human B cells. *Nucleic Acids Res.* 35:e73 10.1093/nar/gkm244 PMC1904266 17478510

[B26] Nakahira K. Kim H. P. Geng X. H. Nakao A. Wang X. Murase N. (2006). Carbon monoxide differentially inhibits TLR signaling pathways by regulating ROS-induced trafficking of TLRs to lipid rafts. *J. Exp. Med.* 203 2377–2389. 10.1084/jem.20060845 17000866 PMC2118097

[B27] O’Neill L. A. J. Golenbock D. Bowie A. G. (2013). The history of Toll-like receptors – redefining innate immunity. *Nat. Rev. Immunol.* 13 453–460. 10.1038/nri3446 23681101

[B28] Otterbein L. E. May A. Chin B. Y. (2005). Carbon monoxide increases macrophage bacterial clearance through Toll-like receptor (TLR)4 expression. *Cell Mol Biol.* 51 433–440. 16309564

[B29] Pae H.-O. Oh G.-S. Choi B.-M. Chae S.-C. Kim Y.-M. Chung K.-R. (2004). Carbon monoxide produced by heme oxygenase-1 suppresses T cell proliferation via inhibition of IL-2 production. *J. Immunol.* 172 4744–4751. 10.4049/jimmunol.172.8.4744 15067050

[B30] Perry S. T. Compton T. (2006). Kaposi’s sarcoma-associated herpesvirus virions inhibit interferon responses induced by envelope glycoprotein gpK8.1. *J. Virol.* 80 11105–11114. 10.1128/JVI.00846-06 16956942 PMC1642153

[B31] Riquelme S. A. Bueno S. M. Kalergis A. M. (2015). Carbon monoxide down-modulates Toll-like receptor 4/MD2 expression on innate immune cells and reduces endotoxic shock susceptibility. *Immunology* 144 321–332. 10.1111/imm.12375 25179131 PMC4298426

[B32] Ruan Y. Wang L. Zhao Y. Yao Y. Chen S. Li J. (2014). Carbon monoxide potently prevents ischemia-induced high-mobility group box 1 translocation and release and protects against lethal renal ischemia-reperfusion injury. *Kidney Int.* 86 525–537. 10.1038/ki.2014.80 24694987

[B33] Rücker H. Amslinger S. (2015). Identification of heme oxygenase-1 stimulators by a convenient ELISA-based bilirubin quantification assay. *Free Radic. Biol. Med.* 78 135–146. 10.1016/j.freeradbiomed.2014.10.506 25462643

[B34] Ryter S. W. Alam J. Choi A. M. K. (2006). Heme oxygenase-1/carbon monoxide: from basic science to therapeutic applications. *Physiol. Rev.* 20 1–68. 10.1152/physrev.00011.2005 16601269

[B35] Ryter S. W. Choi A. M. K. (2009). Heme oxygenase-1/carbon monoxide: from metabolism to molecular therapy. *Am. J. Respir. Cell Mol. Biol.* 41 251–260. 10.1165/rcmb.2009-0170TR 19617398 PMC2742746

[B36] Ryter S. W. Choi A. M. K. (2015). Targeting heme oxygenase-1 and carbon monoxide for therapeutic modulation of inflammation. *Transl. Res.* 16 1–28. 10.1016/j.trsl.2015.06.011 PMC4857893 26166253

[B37] Ryter S. W. Tyrrell R. M. (2000). The heme synthesis and degradation pathways: role in oxidant sensitivity. Heme oxygenase has both pro- and antioxidant properties. *Free Radic. Biol. Med.* 28 289–309. 10.1016/S0891-5849(99)00223-3 11281297

[B38] Sathish N. Yuan Y. (2011). Evasion and subversion of interferon-mediated antiviral immunity by Kaposi’s sarcoma-associated herpesvirus: an overview. *J. Virol.* 85 10934–10944. 10.1128/JVI.00687-11 21775463 PMC3194983

[B39] Schneider W. M. Chevillotte M. D. Rice C. M. (2014). Interferon-stimulated genes: a complex web of host defenses. *Annu. Rev. Immunol.* 32 513–545. 10.1146/annurev-immunol-032713-120231 24555472 PMC4313732

[B40] Schoggins J. W. Rice C. M. (2011). Interferon-stimulated genes and their antiviral effector functions. *Curr. Opin. Virol.* 1 519–525. 10.1016/j.coviro.2011.10.008 22328912 PMC3274382

[B41] Sheedy F. J. Palsson-McDermott E. Hennessy E. J. Martin C. O’Leary J. J. Ruan Q. (2009). Negative regulation of TLR4 via targeting of the proinflammatory tumor suppressor PDCD4 by the microRNA miR-21. *Nat. Immunol.* 11 141–147. 10.1038/ni.1828 19946272

[B42] Singh V. V. Dutta D. Ansari M. A. Dutta S. Chandran B. Longnecker R. (2014). Kaposi’s sarcoma-associated herpesvirus induces the ATM and H2AX DNA damage response early during *de novo* infection of primary endothelial cells, which play roles in latency establishment. *J. Virol.* 88 2821–2834. 10.1128/JVI.03126-13 24352470 PMC3958070

[B43] Tsiftsoglou A. S. Tsamadou A. I. Papadopoulou L. C. (2006). Heme as key regulator of major mammalian cellular functions: molecular, cellular, and pharmacological aspects. *Pharmacol. Ther.* 111 327–345. 10.1016/j.pharmthera.2005.10.017 16513178

[B44] Wang J. Karpus J. Zhao B. S. Luo Z. Chen P. R. He C. (2012). A selective fluorescent probe for carbon monoxide imaging in living cells. *Angew Chem. Int. Ed. Engl.* 51 9652–9656. 10.1002/anie.201203684 22930547

[B45] Wang X. M. Kim H. P. Nakahira K. Ryter S. W. Choi A. M. K. (2009). The heme oxygenase-1/carbon monoxide pathway suppresses TLR4 signaling by regulating the interaction of TLR4 with caveolin-1. *J. Immunol.* 182 3809–3818. 10.4049/jimmunol.0712437 19265160

[B46] West J. Damania B. (2008). Upregulation of the TLR3 pathway by Kaposi’s sarcoma-associated herpesvirus during primary infection. *J. Virol.* 82 5440–5449. 10.1128/JVI.02590-07 18367536 PMC2395190

[B47] West J. A. Gregory S. M. Sivaraman V. Su L. Damania B. (2011). Activation of plasmacytoid dendritic cells by Kaposi’s sarcoma-associated herpesvirus. *J. Virol.* 85 895–904. 10.1128/JVI.01007-10 20980519 PMC3020034

[B48] Xue J. Habtezion A. (2014). Carbon monoxide-based therapy ameliorates acute pancreatitis via TLR4 inhibition. *J. Clin. Invest.* 124 437–447. 10.1172/JCI71362 24334457 PMC3871246

[B49] Yang Y.-C. Huang Y.-T. Hsieh C.-W. Yang P.-M. Wung B.-S. (2014). Carbon monoxide induces heme oxygenase-1 to modulate STAT3 activation in endothelial cells via S-glutathionylation. *PLoS ONE* 9:e100677 10.1371/journal.pone.0100677 PMC4114553 25072782

