# Supplementary material for: The Heme Metabolite Carbon Monoxide Facilitates KSHV Infection by Inhibiting TLR4 Signaling in Endothelial Cells
Source: Front Microbiol. 2017 Apr 3;8:568. doi: 10.3389/fmicb.2017.00568 (PMC5376558; doi:10.3389/fmicb.2017.00568)
Supplement: Supplementary file 1 [file Presentation_1.PPTX]

## Slide 1
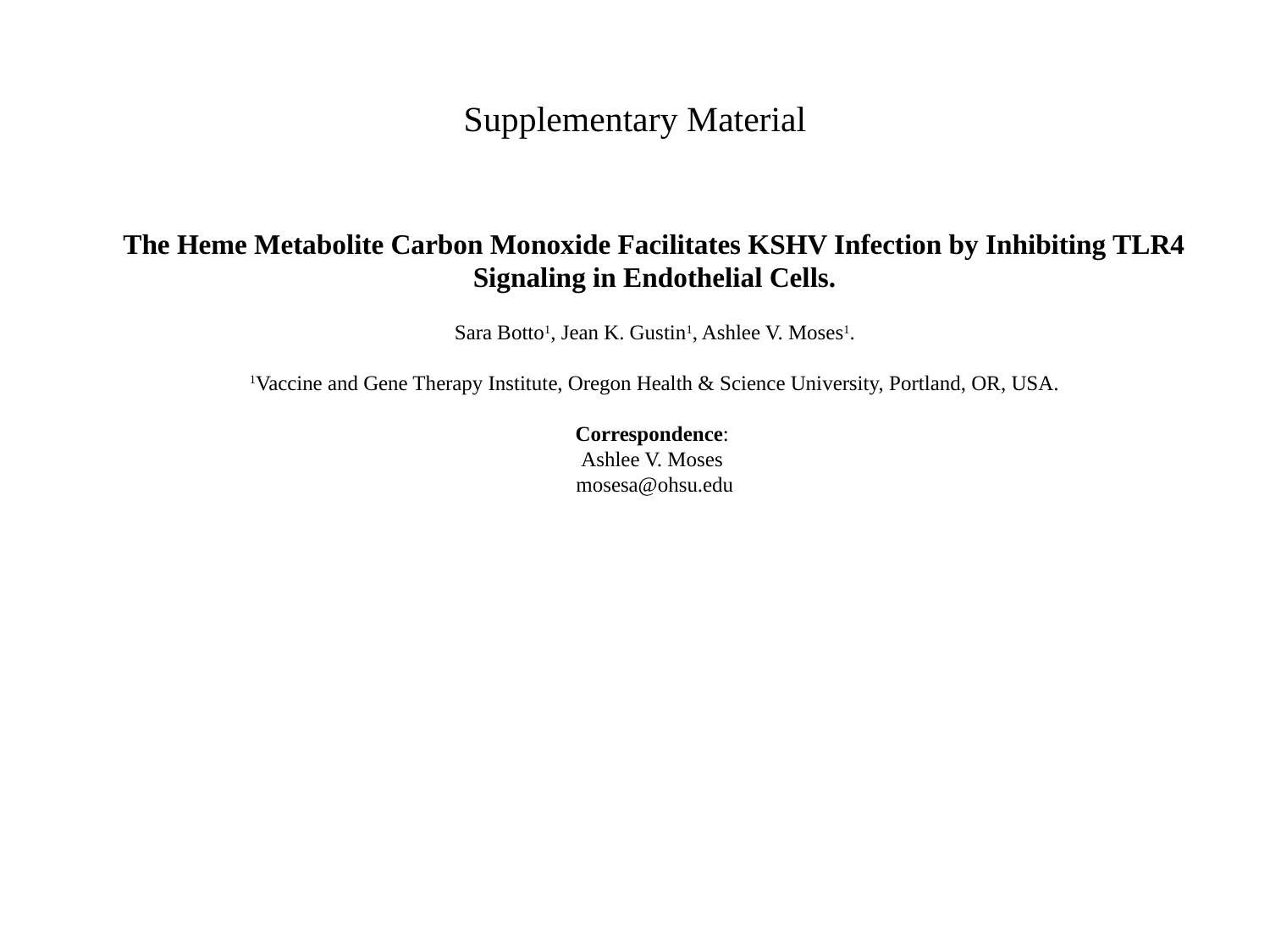

# Supplementary Material
The Heme Metabolite Carbon Monoxide Facilitates KSHV Infection by Inhibiting TLR4 Signaling in Endothelial Cells.
Sara Botto1, Jean K. Gustin1, Ashlee V. Moses1.
1Vaccine and Gene Therapy Institute, Oregon Health & Science University, Portland, OR, USA.
Correspondence:
Ashlee V. Moses
mosesa@ohsu.edu

## Slide 2
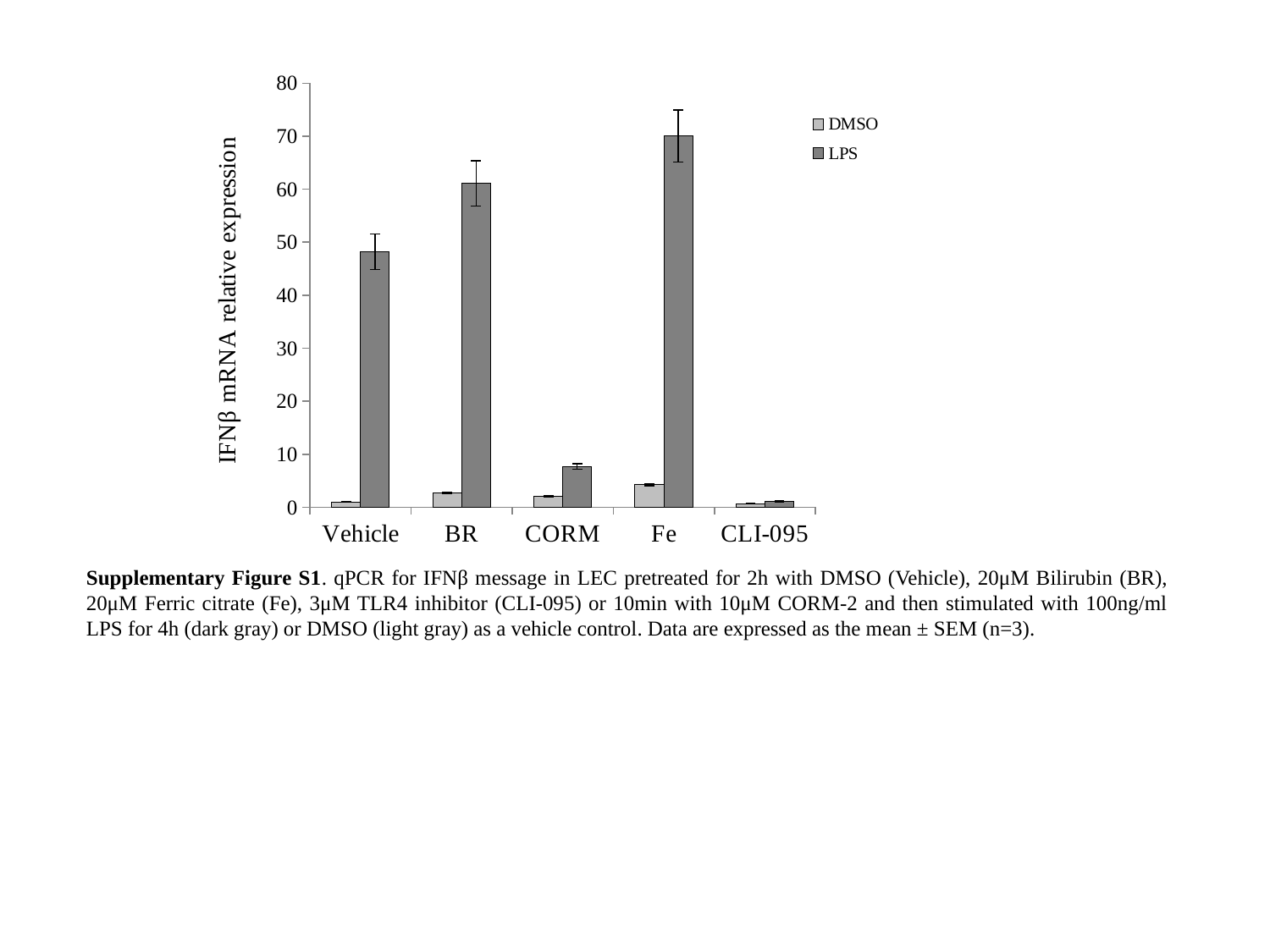

### Chart
| Category | | |
|---|---|---|
| Vehicle | 1.0 | 48.2155999580446 |
| BR | 2.741945697999064 | 61.09481726675349 |
| CORM | 2.016285591391435 | 7.689021428517494 |
| Fe | 4.245545693848037 | 70.0191719263 |
| CLI-095 | 0.672264093257477 | 1.098473449711355 |Supplementary Figure S1. qPCR for IFNβ message in LEC pretreated for 2h with DMSO (Vehicle), 20μM Bilirubin (BR), 20μM Ferric citrate (Fe), 3μM TLR4 inhibitor (CLI-095) or 10min with 10μM CORM-2 and then stimulated with 100ng/ml LPS for 4h (dark gray) or DMSO (light gray) as a vehicle control. Data are expressed as the mean ± SEM (n=3).

## Slide 3
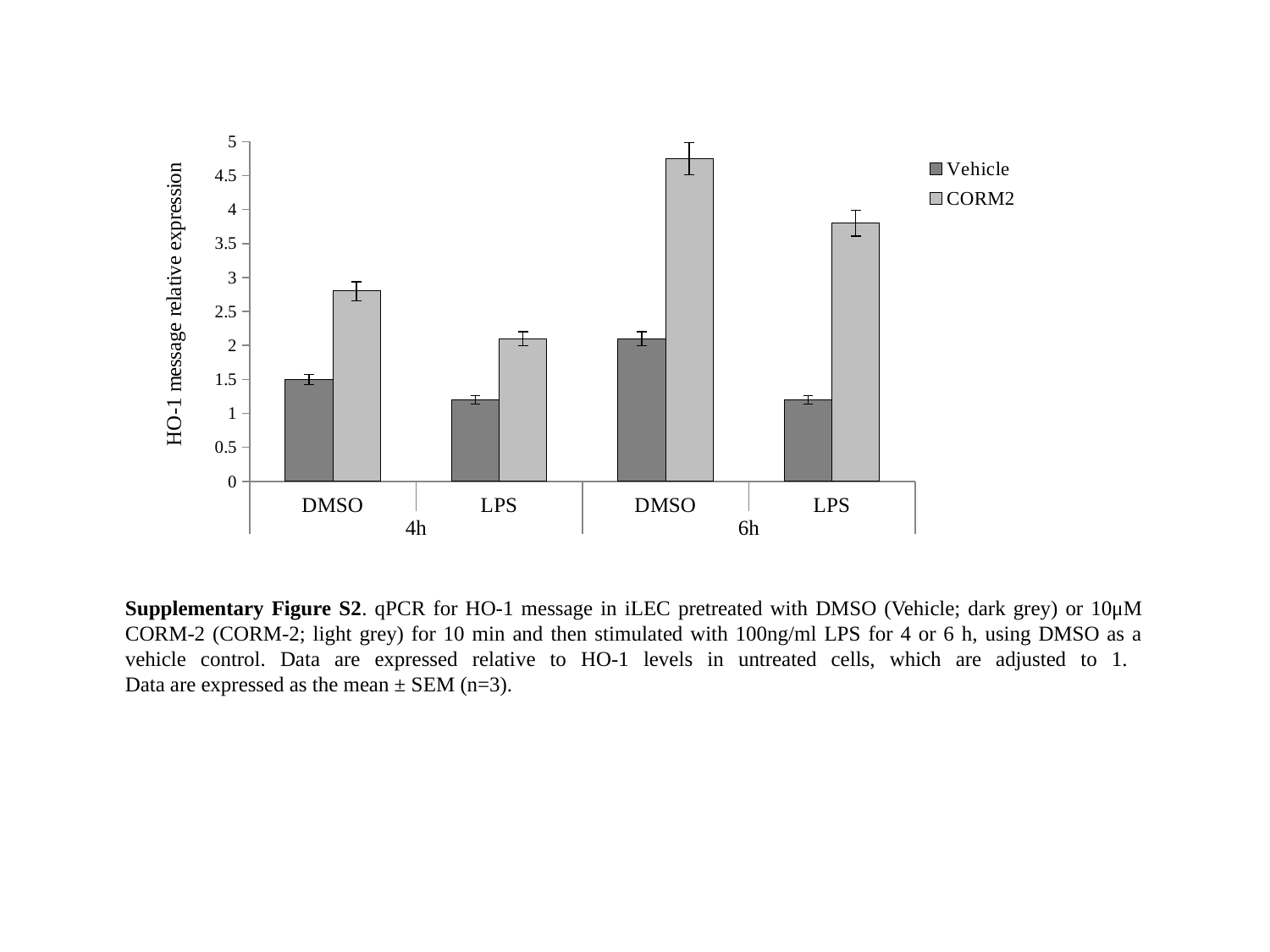

### Chart
| Category | | |
|---|---|---|
| DMSO | 1.5 | 2.8 |
| LPS | 1.2 | 2.1 |
| DMSO | 2.1 | 4.75 |
| LPS | 1.2 | 3.8 |Supplementary Figure S2. qPCR for HO-1 message in iLEC pretreated with DMSO (Vehicle; dark grey) or 10μM CORM-2 (CORM-2; light grey) for 10 min and then stimulated with 100ng/ml LPS for 4 or 6 h, using DMSO as a vehicle control. Data are expressed relative to HO-1 levels in untreated cells, which are adjusted to 1.
Data are expressed as the mean ± SEM (n=3).

## Slide 4
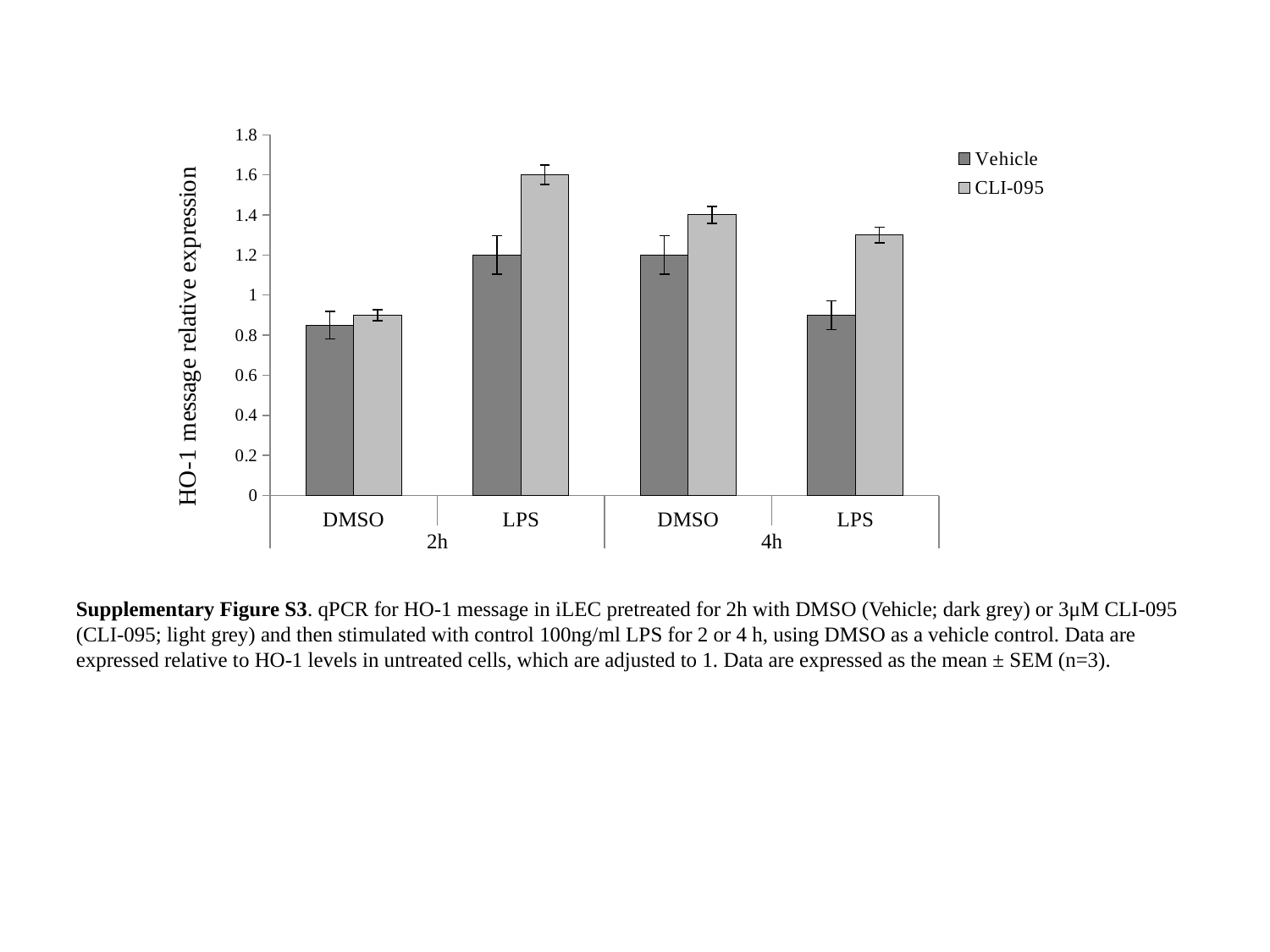

### Chart
| Category | | |
|---|---|---|
| DMSO | 0.85 | 0.9 |
| LPS | 1.2 | 1.6 |
| DMSO | 1.2 | 1.4 |
| LPS | 0.9 | 1.3 |Supplementary Figure S3. qPCR for HO-1 message in iLEC pretreated for 2h with DMSO (Vehicle; dark grey) or 3μM CLI-095 (CLI-095; light grey) and then stimulated with control 100ng/ml LPS for 2 or 4 h, using DMSO as a vehicle control. Data are expressed relative to HO-1 levels in untreated cells, which are adjusted to 1. Data are expressed as the mean ± SEM (n=3).

## Slide 5
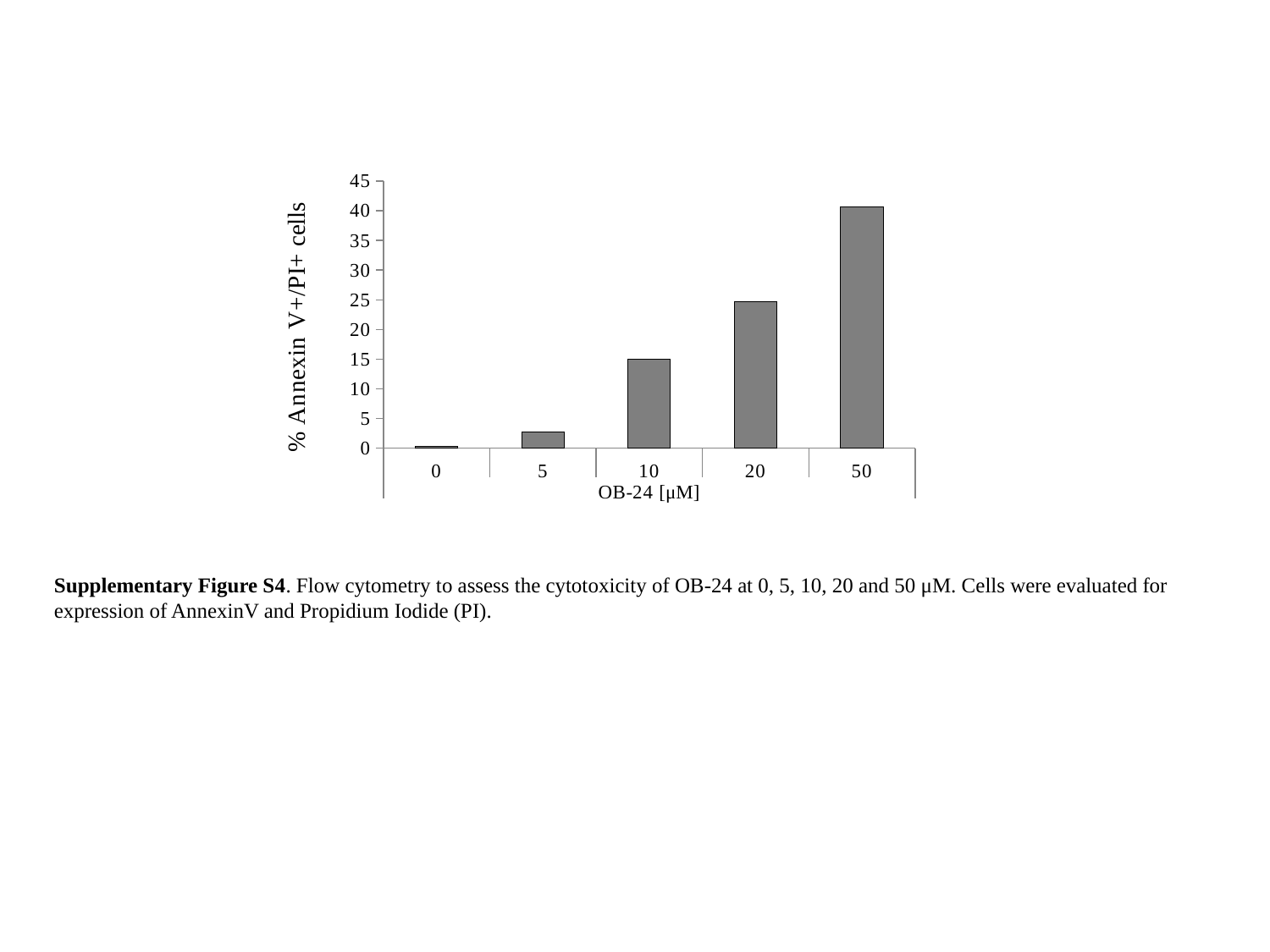

### Chart
| Category | |
|---|---|
| 0 | 0.268 |
| 5 | 2.7 |
| 10 | 15.0 |
| 20 | 24.7 |
| 50 | 40.7 |Supplementary Figure S4. Flow cytometry to assess the cytotoxicity of OB-24 at 0, 5, 10, 20 and 50 μM. Cells were evaluated for expression of AnnexinV and Propidium Iodide (PI).
